# The Communication between the PI3K/AKT/mTOR Pathway and Y-Box Binding Protein-1 in Gynecological Cancer

**DOI:** 10.3390/cancers12010205

**Published:** 2020-01-14

**Authors:** Monika Sobočan, Suzana Bračič, Jure Knez, Iztok Takač, Johannes Haybaeck

**Affiliations:** 1 Department of Pharmacology, Faculty of Medicine, University of Maribor, 2000 Maribor, Slovenia; 2 Divison of Gynecology and Perinatology, University Medical Centre Maribor, 2000 Maribor, Slovenia; knez.jure@gmail.com (J.K.); iztok.takac@ukc-mb.si (I.T.); 3 Department of Obstetrics and Gynecology, Faculty of Medicine, University of Maribor, 2000 Maribor, Slovenia; 4 Department of Pathology, Hospital Graz II, West, 8020 Graz, Austria; suzy.bracic@gmail.com; 5 Department of Pathology, Faculty of Medicine, University of Maribor, 2000 Maribor, Slovenia; 6 Department of Pathology, Medical Faculty Otto-von-Guericke University Magdeburg, 39120 Magdeburg, Germany; johannes.haybaeck@med.ovgu.de; 7 Department of Pathology, Neuropathology and Molecular Pathology, Medical University of Innsbruck, 6020 Innsbruck, Austria; 8 Diagnostic and Research Institute of Pathology, Medical University of Graz, 8036 Graz, Austria

**Keywords:** mTOR signaling, YB-1, endometrial cancer, ovarian cancer, cervical cancer

## Abstract

Studies of the mechanistic (mammalian) target of rapamycin inhibitors (mTOR) represent a step towards the targeted treatment of gynecological cancers. It has been shown that women with increased levels of mTOR signaling pathway targets have worse prognosis compared to women with normal mTOR levels. Yet, targeting mTOR alone has led to unsatisfactory outcomes in gynecological cancer. The aim of our review was therefore to provide an overview of the most recent clinical results and basic findings on the interplay of mTOR signaling and cold shock proteins in gynecological malignancies. Due to their oncogenic activity, there are promising data showing that mTOR and Y-box-protein 1 (YB-1) dual targeting improves the inhibition of carcinogenic activity. Although several components differentially expressed in patients with ovarian, endometrial, and cervical cancer of the mTOR were identified, there are only a few investigated downstream actors in gynecological cancer connecting them with YB-1. Our analysis shows that YB-1 is an important player impacting AKT as well as the downstream actors interacting with mTOR such as epidermal growth factor receptor (EGFR), Snail or E-cadherin.

## 1. Introduction

Malignancies of the endometrium, cervix, and ovaries represent the most common gynecological malignancies. It is a particular challenge to treat these cancers at advanced disease stages or at the time of recurrence. Several attempts have already been made to target signaling pathways in order to improve the overall survival (OS) and progress-free survival (PFS) of women with gynecological malignancies. One approach towards targeted treatment is the use of a mechanistic (mammalian) target of rapamycin (mTOR) inhibitors [1]. The current level of knowledge is that the outcomes for women with gynecological cancer and elevated levels of mTOR signaling pathway targets seem to be significantly worse than those for women with normal mTOR levels [2]. Significantly increased levels of mTOR resulted in poorly differentiated tumors and correlated positively with lymph node involvement. If nuclear mTOR expression was increased, the PFS rate was also lower. However, the overexpression of mTOR or protein kinase B (more commonly referred to as Akt) was not associated with specific histological subtype features of the tumor [3]. This led to the development and clinical testing of mTOR inhibitors in gynecological cancers. However, in gynecological cancers, the success of the clinical trials was limited [1]. Therefore, an investigation of different processes involved in tumor development and progression interacting with the mTOR pathway is warranted. One possibility of such an interaction is the interplay between cold shock proteins, one of the most evolutionary conserved proteins [4], and the mTOR pathway. This review addresses recent advances made in the study of aberrations and triggers of the mTOR signaling pathway in gynecological malignancies in the context of their interaction with cold shock proteins, thus allowing us to better understand the stratification of patients undergoing mTOR therapy and future disease prediction.

Cold shock proteins (CSP) are a family of proteins where Y-box binding protein-1 (YB-1) is one of the best characterized proteins [5]. An important feature of CSP is their presence in one or more cold shock domains (CSD). The domains possess nucleic acid binding properties and thus function pleiotropically in processes such as transcription, translation, and cell proliferation [4,6]. In humans, YB-1 represents the predominant form of CSP. It functions in the nucleus as a transcription factor as well as in the cytoplasm where it directly interacts with mRNA splicing and the storage of mRNA. By binding to internal ribosomal entry sites (IRES), YB-1 impacts the translation in Snail, Twist, and HIF1alpha and effects the epithelial mesenchymal transition (EMT) [6]. Recent studies in colorectal cancer showed that combining strategies of silencing a dual target, YB-1 through the inhibition of p90 ribosomal S6 kinase (RSK) and Akt, lead to improved sensitivity to standard systemic therapy [7]. This warrants the attempt to connect and understand the impactful molecular mechanisms behind the signaling. 

## 2. Clinical Investigation into mTOR Signaling and Inhibition in Women with Gynecological Cancers

### 2.1. Endometrial Cancer

Endometrial cancer (EC) is the most frequently diagnosed gynecological malignancy [8]. It mostly consists of type I tumors, characterized as endometrioid adenocarcinomas or, in rare occasions, as mucinous carcinomas. These tumors are low-grade and estrogen-related tumors. This is in contrast to type II non-endometrioid carcinomas, which are histologically serous or clear cell carcinomas, and are unrelated to estrogen. Type II EC also behaves more aggressively [8,9]. To stratify patients, one of the emerging markers connecting a personalized prognosis for women with endometrial EC is the presence of the nuclear p-Ser167-Era, an estrogen receptor that interacts with the mitogen-activated protein kinase (MAPK) and mTOR pathway. Patients with positively stained p-Ser167-Era had a shorter recurrence-free survival (RFS), and p-Ser167-Era was positively correlated with pS6K1 and pMAPK staining [10]. Against the background of a growing understanding of mTOR signaling, trials investigating mTOR inhibitors in EC have been conducted in recent years. The inhibition of mTOR was used as monotherapy or as a combination therapy with other substances, whose focus is the determination of different targets in the mTOR signaling pathway. The most common therapy trials in EC are carried out using mammalian target of rapamycin complex 1 (mTORC1) inhibitors (temsirolimus, everolimus) as well as mTORC1/2 inhibitors (AZD2014), AKT inhibitors (AZD5363, MIK2206, GSK2141795), and dual mTOR/AKT inhibitors (MKC1) [11]. Interestingly very scarce literature is available on the expression of YB-1 in endometrial tissue or EC. Silvera et al. [12] found, that, in patients with endometriosis, in comparison to patients without endometriosis, there are high levels of YB-1 expression present. A knockdown of YB-1 resulted in cell growth inhibition, a cessation of YB-1 proliferation and an increase in apoptosis [12]. To the best of our knowledge, there is currently no published research investigating the role ob YB-1 and mTOR signaling in EC.

### 2.2. Cervical Cancer

Cervical cancer (CC) is the fourth leading cause of cancer-related deaths in women. Although the number of deaths caused by CC has decreased, especially in countries implementing screening programs, the prognosis for patients affected by this disease is still poor [13]. The different CC subtypes show significant differences as regards the clinical aspects and biology. Clinically, squamous cell carcinomas (SCC) account for the majority of cervical tumors. As far as their incidence is concerned, adenocarcinomas in CC follow in second place. They account for approximately 10–20% of all CC cases, and their incidence is increasing [9]. Considering its development, CC is a multifactorial disease related to HPV (human papilloma virus), tumor suppressor genes, oncogenes, and others [14]. It is driven by different molecular mechanisms that are not yet fully understood. The mTOR signaling pathway is assumed to be one of those mechanisms. A study investigating mTOR signaling of cervical cancer cell lines revealed high mTOR activity in CC [15]. Immunohistochemical analysis of the mTOR pathway markers phosphorylated-mTOR (p-mTOR), p-p70S6K, and EGFR revealed a highly positive expression in high grade squamous intraepithelial lesions (HSIL) and all SCC stains. Especially SCCs showed increased nuclear translocation of p-mTOR and p-p70S6K in all cases and in most HSIL cases [16]. A few trials have evaluated the value of mTOR inhibitors in CC. These trials used mTORC1 inhibitors (everolimus or trials including temsirolimus) [17]. Nishio et al [5] explored YB-1 overexpression in cervical cancer. It was shown, that YB-1 expression correlated with epidermal growth factor receptor (EGFR) overexpression, human epidermal growth factor receptor 2 (HER2) overexpression, and worse OS [5]. Research on the clinical impact of YB-1 showed that, in patients with overexpressed YB-1, chemoradiosensitivity was decreased [18]. This might be due to the interaction of YB-1 with HPV18 E6 mRNA regulation [19].

### 2.3. Ovarian Cancer 

Different subtypes and clinicopathological features are even more evident in ovarian cancer (OC) [9,20]. The classification of ovarian carcinomas is based on the histological differentiation of the tumors according to the World Health Organization (WHO). Tumors are divided into epithelial, sex cord-stromal, and germ cell neoplasms. The tumors with the highest incidence of ovarian carcinoma are epithelial ovarian tumors. They are subdivided into serous, mucinous, endometrioid, clear cell and transitional cell tumors (inclusive of Brenner tumors) [20]. The understanding of carcinogenesis in OC is currently based on a model that divides OC in two groups: (i) Type I: low-grade serous, endometrioid, clear cell, mucinous carcinoma, and Brenner tumors and (ii) Type II: high grade serous, high-grade endometrioid, undifferentiated carcinomas, and mixed mesodermal tumors. The slow-growing type I tumors can turn into Type II tumors over time [20]. As in endometrial cancer, targets of mTOR inhibitors in ovarian carcinoma are in most cases the mTORC1 complex, as well as the mTORC1/2 complex inhibitor in combination with AKT inhibition [11]. Molecular studies found no specific mTOR target that pointed to statistically significant clinical outcomes for patients treated with common mTOR inhibitors IND160A and IND160B (temsirolimus) as well as IND192 (ridaforolimus) [21]. YB-1 has been proposed to be an oncogene for OC for more than a decade. It has been connected to promote tumor growth and multi drug resistance through interaction with genes and growth factors [12]. In exploring the possibility of determining YB-1 in serum samples, it was further shown that a decreased level of YB-1/p18 correlated with ovarian cancer compared to the control group of healthy volunteers. This initially contradictory data can be understood as the formation of homodimers and multimer-formation in the extracellular space, and as such leads to lower levels of YB-1/p18 in OC patients [22]. 

### 2.4. Ongoing Clinical Trials 

Current ongoing trials investigating the role of different mTOR pathway inhibitors are depicted in Table 1. The current clinical trials use the knowledge on signaling pathways in gynecological cancer and combine inhibition in most cases of at least two pathways. This is particularly illustrated in NCT01596140 where the trial is investigating the inhibition of the B-Raf/MEK step on the mitogen activated protein kinase/extracellular signal-related kinase (B-Raf/MEK/ERK) pathway through vemurafenib and the mTOR pathway through everolimus or temsirolimus. Some clinical trials have moved to the investigation of molecular targeted therapy for mTOR using the alterations in the mTOR signaling pathway as landmarks for patient selection (e.g., NCT02029001 or NCT02465060). However, to our current knowledge there are no clinical trials investigating YB-1 as a marker in gynecological cancer. However, knowledge is emerging that momelotinib (CYT387), a pharmacological inhibitor of noncanonical IkB kinase/TANK-binding kinase 1 (IKBKE/TBK1) which is used in acute myeloid leukemia, might be able to regulate tumor MYC-dependent survival through YB-1 [9,23]. This opens new pathways of investigation for the enhancement of current mTOR inhibition or YB-1 regulation. 

## 3. The PI3K/AKT/mTOR Impact on Molecular Pathophysiology of Gynecological Cancer

### 3.1. PI3K/AKT/mTOR Pathway

The phosphoinositide 3-kinases/protein kinase B/mammalian target of rapamycin (PI3K/AKT/mTOR) pathway is a signaling pathway long known to be present in the normal cell physiology and oncogenesis [2]. It regulates cell growth and cell cycle progression through growth stimuli and signal integration [9]. The cascade is frequently hyperactivated in several cancer subtypes [24]. Factors dysregulating the pathway, such as increased growth factor stimuli, mutation activation, loss of gene function, or kinase gene activation, contribute to aberrant signaling, leading to carcinogenic events [9]. Studies have shown that the dysregulation of mTOR pathway influences the process of carcinogenesis in many cancers, such as ovarian cancer, lung cancer, prostate cancer, and mantle cell lymphoma [15]. 

The PI3K/AKT/mTOR pathway consists of three driving molecules: (i) PI3K, a member of the lipid kinase family that phosphorylates the 3-hydroxyl group of phosphoinositide; (ii) AKT, a serine/threonine kinase; and (iii) the mTOR [2]. The mechanism of action starts with the stimulation of the PI3K through growth factors, which leads to activation of the cascade by the phosphorylation of phosphatidylinositol-4,5-bis-phosphate (PIP2) to develop phosphatidylinositol-3,4,5-triphosphate (PIP3). Once PIP3 is accumulated, the cascade events start to unfold. Activation starts by the phosphorylation of AKT through phosphoinositide-dependent kinase-1 (PDK1) [9]. AKT then phosphorylates and inhibits the tuberous sclerosis complex (TSC), influencing the inhibition effect of Ras-related small GTPase Rheb (Ras-homolog-enriched-in-brain). This leads to the positive up-stream regulation of mTOR, which is an atypical serine/threonine kinase that belongs to the PIKK (phosphoinositide 3-kinase related protein kinase) family [9,25]. Only one mTOR gene has been identified in mammalian cells. mTOR is activated in coordination with other protein complexes [6,7]. It functions as a catalytic subunit of two protein kinase complexes, referred to as mTORC1 and mTORC2 [26]. mTORC1 activation is associated with the Regulatory-associated protein of mTOR (RAPTOR), the regulatory-associated protein of mTOR. This leads to the phosphorylation of the eukaryotic translation initiation factor (4E-BP1) and the ribosomal protein kinase 1 (S6K1). Downstream, the mechanism of action therefore initiates protein synthesis, such as cell cycle proteins, vascular endothelial growth factor (VEGF), or c-Myc [9]. The complex mTORC2 is different from mTORC1 since it is not sensitive to rapamycin. It participates in cell survival and proliferation by its ability to control AKT activity through the phosphorylation of AKT at serine-473 [2]. 

### 3.2. mTORC1 and mTORC2 Complexes in Gynecological Malignancies 

Montero et al. [27] investigated the function of mTORC1 and mTORC2 in ovarian cancer cell proliferation. Through the knockdown of RAPTOR or rapamycin-insensitive companion of mammalian target of rapamycin (RICTOR), they found that, compared to mTORC2, mTORC1 played a predominant role in ovarian cancer. The inhibition was visible on the level of S6 phosphorylation, a marker of mTORC1 function [27]. Interestingly, rapamycin had a small effect on 4E-BP1, another substrate of mTORC1 [27]. Noske et al. then further investigated [28] the overexpression of p-eIF-4E (56%) in primary ovarian carcinomas in a comparison to borderline tumors. They found that p-mTOR expression correlated with p-eIF-4E and the serous histological type. The increased p-mTOR and p-eIF-4E values were correlated with a higher mitotic rate (*p* = 0.004) and with poor differentiation (*p* = 0.04). Recent studies have tried to gain a deeper understanding of the role of mTORC2 in carcinogenesis. Liu et al. therefore explored the role of Sin1 in mTORC2 complex and found that phosphorylation of AKT or S6K was a prerequisite for the Sin1 activation of mTORC2 [29]. Especially 4E-BP seemed promising as a marker for mTOR signaling inhibition as it was previously implied, that 4EBP and p70S6 kinase communicate and inhibit the oncogene YB-1. However, studies by Lyabin and Ovchinnikov [30] on the inhibition of YB-1 synthesis through rapamycin showed, that inhibition produced no effect on YB-1 synthesis in CC cell lines as well as no effect on translation. It was suggested that previously visible inhibition of YB-1 mRNA was not specifically due to targeted interaction on 4E-BP, but to a dependence of YB-1 mRNA on the eIF4F-group factors. Thus, once inhibition targeted eIF4F and impacted mRNA availability, this also downregulated YB-1 mRNA [30].

### 3.3. Metabolic Impact on Carcinogenesis by mTOR 

Using a well-established phosphatase and tensin homologue (PTEN) knockout model, Iglesias and colleagues found that in endometrial hyperplasia (EH), obesity or lean body weight in mouse models did not affect the development of EH. There was no significant difference between lean and obese mice in the progression of normal endometrium to EH, nor were there any significant differences in the alteration of the mTOR pathway [31]. Studies have associated higher body mass index (BMI) with EC for a long time. To understand this mechanism on a molecular level, Sahoo and colleagues examined the effects of adipocyte-conditioned medium (ACM) on EC cell lines in a comparison with pre-adipocyte-conditioned medium (PACM). They found that in ACM, the vascular endothelial growth factor (VEGF), a downstream signaling pathway connected to the mTOR pathway, was upregulated. This correlates with the finding that mTOR pathway upregulation leads to larger EC tumors [32]. Zhu et al. report that the clinical outcome of EC is influenced by the expression of fat mass and the obesity-associated gene (FTO). Using immunohistochemistry and Western blotting, they observed that obese women with endometrial cancer showed estrogen (ERα-dependent)-induced FTO nuclear accumulation in the mTOR signaling pathway, which contributed to worse clinical outcomes [33]. In vitro tests carried out to determine the cell response to glucose in terms of cytotoxicity, apoptosis, cell cycle and adhesion/invasion revealed increased cell growth in the presence of high glucose levels. The behavior of EC cells was dependent on the level of glucose and influenced adhesion and invasion. E-Cadherin expression was decreased, while Snail expression was increased. The adenosine monophosphate-activated protein kinase/(AMPK)/mTOR/S6 and mitogen-activated protein kinase (MAPK) pathway was affected by upregulation of glucose metabolism [34]. A downregulation of E-cad with a Snail upregulation is considered to be a hallmark of EMT [19]. 

### 3.4. Carcinogenesis in Presence and Absence of HPV

**Figure 1:**
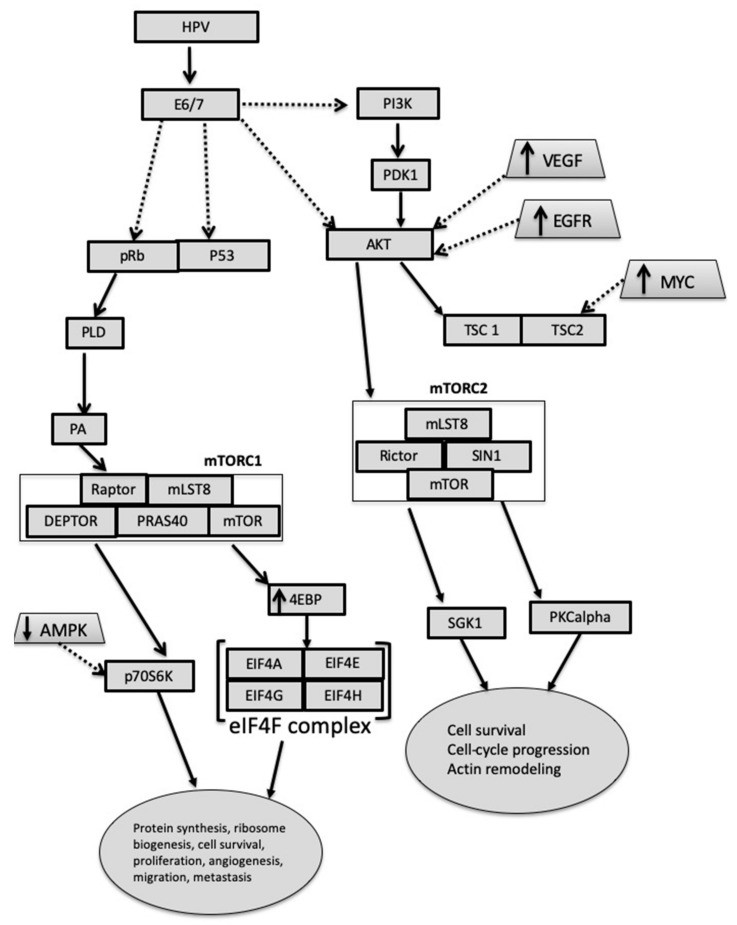
A simplistic model of the mTOR signaling pathway in cervical cancer. Abbreviations: EIF: eukaryotic initiation factor, AMPK: adenosine monophosphate-activated protein kinase, PKCalpha: Protein kinase C-alpha, SGK1: serum and glucocorticoid-regulated kinase 1, 4EBP: Eukaryotic translation initiation factor 4E-binding protein 1, mTOR: mammalian target of rapamycin, SIN1: mammalian stress-activated protein kinase interacting protein 1, Rictor: Rapamycin-insensitive companion of mammalian target of rapamycin, mTORC1: rapamycin-sensitive mTOR-Raptor, mTORC2: rapamycin-insensitive mTOR-Rictor, mLST8: target of rapamycin complex subunit LST8, Raptor: regulatory-associated protein of mTOR, PRAS40: roline-rich Akt substrate of 40 kDa, PA: phosphatidic acid, PLD: phospholipase D, pRb: retinoblastoma protein, HPV: Human Papilloma Virus, PI3K: phosphoinositide 3-kinase, PDK1: Pyruvate Dehydrogenase Kinase 1, Akt: Protein kinase B, VEGF: Vascular endothelial growth factor, EGFR: epidermal growth factor receptor, TSC: Tuberous sclerosis 1.

It is increasingly recognized that HPV oncoproteins E6 and E7 induce the process of carcinogenesis not only by affecting p53 and pRb, but also by molecular events in mTOR signaling [35]. In Figure 1, the cascade of mTOR signaling in coordination with the human papilloma virus (HPV) E6 is shown. This protein expression was shown by Pang et al [19] to regulate the levels of mRNA expression of YB-1 and by this promoting the progression of CC: The mechanism is based on the observations that E6 promoted enhanced expression of Snail [19]. Further on, studies investigating oncogenic protein RAS show that by the background expression of E6 E7, transformation of human cervical keratinocytes (HCK) is initiated, and that MYC and HRAS-oncogene are critical stakeholders in tumorigenic transformation. Increased levels of MYC lead to an increase in survivin and p-4EBP1 levels, as well as in p70S6K levels, but to decreased Tuberous Sclerosis Complex 2 (TSC2) levels [36]. Molinolo et al. compared the expression of pS6 and Akt^S473^ in HPV-positive and HPV-negative oral and cervical squamous cancers. They found that while stable HPV infections had no particular impact on proliferation, the presence of p16, a staining marker for the E6 HPV oncoprotein, triggered the degradation of TSC2, a downstream target of mTORC1. Further investigations then showed that mTOR inhibitors had a particular effect on mTORC1 signaling pathway in SCC, but access to the mTORC2 pathway was limited.

Studies using a model of LKB1 deficiency have shown that carcinogenesis is influenced not only by HPV oncogene initiation of mTOR pathway overexpression, but also plays an important role in the initiation of the pathway [37]. Furthermore, phosphoacidic acid (PA), a product of phospholipase D (PLD), has been shown to be required for the activation of mTORC1 through mitogen and amino acid signaling, and to be able to intervene independently with DEP Domain Containing MTOR Interacting Protein (DEPTOR), the mTOR protein for mTORC1 and mTORC2 inhibition. This cascade of E7-based initiation of carcinogenesis is known to contribute to AKT activation in in vivo experiments [38].

A smaller proportion of CC pathologies are unrelated to the presence of high-risk HPV. A histological subtype unrelated to the HPV carcinogenesis initiation is the clear cell cervical carcinoma (CCCC). A small study investigated the impact of autophosphorylation of EGFR, HER2, p-mTOR, and pAKT and its contribution to downstream pathways of the four core protein kinases (RAS/RAF/MEK/ERK) and to the PI3K/AKT/mTOR pathway. All cases of CCCC showed increased expression of EGFR, HER2, AKT or mTOR [39]. This is especially interesting as the overexpression of EGFR and HER2 was associated with YB-1 overexpression enabling us to further hypothesize the underlying linkage between HPV and YB-1. However, mTOR signaling activation was also shown to be activated in the absence of E6 or p53 under the influence of the transcriptional factor PPARbeta. It was shown that PPARbeta acts as an agonist for VEGF in CC cell lines and did not require target nuclear receptors for higher levels of VEGF mRNA [40].

### 3.5. Growth Factors in mTOR Dysregulation

**Figure 2:**
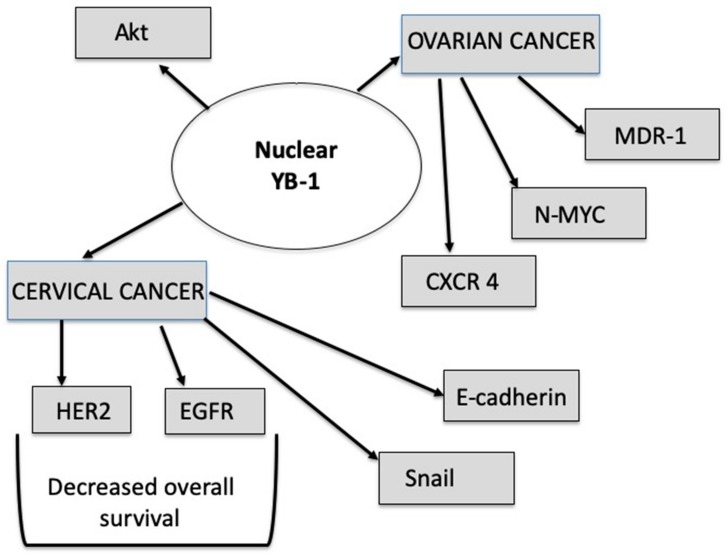
Identified downstream targets of YB-1 in gynecological cancer. Abbreviations: YB-1: Y box binding protein 1, HER2: human epidermal growth factor receptor 2, EGFR: epidermal growth factor receptor, CXCR4: chemokine receptor type 4, MDR-1: multidrug resistance mutation 1, AKT: Protein kinase B.

A group of effectors triggering gynecological cancer development through the mTOR pathway can be identified as growth factors. Especially the epidermal growth factor receptor (EGFR) overexpression has been connected to a more aggressive tumor behavior [41]. Nishio et al [5] reported especially an important correlation of the EGFR overexpression with YB-1 expression in cervical cancer. As depicted in Figure 2, the overexpression of EGFR was connected with worse OS in patients. Additionally, VEGF (vascular endothelial growth factor) [42], IGF (insulin like growth factor) [43], GDF-15 (growth differentiation factor 15) [44] and CYR61 (cysteine rich protein 61) were involved in influencing the mTOR signaling pathway [45].

Early investigations on the role of VEGF in epithelial OC showed that the VEGF-A receptor (VEGFR2) upregulation especially was connected with the overexpression of pS6. The interplay between VEGFR2 activation and pS6 activation led to an increased incidence of ascites, and reduced the response to the standard chemotherapy [42]. By inhibiting the mTOR pathway, Chen et al. found that, in CC, VEGF-A overexpression correlated with cell growth through cyclinD1, CDK4 activation, and invasion through Matrix Metallopeptidase 2 (MMP2) and MMP3 [46]. 

An investigation on Insulin-like growth factor 1 (IGF 1) revealed elevated levels in women with OC. Lau and Leung [43] then further investigated the mechanism of action to answer the question of how tumor progression is possible through the involvement of IGF1. They found that the mTOR signaling pathway is necessary for IGF-1-affected E-cadherin down-regulation [43], revealing a novel mechanism of action of IGF-1 in OC carcinogenesis. Conducting carboplatin-induced apoptosis studies, Lee et al. [45] reported overexpression of CYR61 growth factor, a member of the CCN protein family, in OC. Using that model, CYR61 was found to promote cell proliferation and to inhibit apoptosis. Carboplatin-induced apoptosis cells showed increased AKT phosphorylation, which was not the case in the rapamycin-treated group [45]. 

Studies on the impact of EGF on EMT in a model of endometrioid ovarian carcinoma revealed that EGF induction activated AKT and reduced levels of expressed PTEN, but did not increase cell proliferation. This was assumed to be due to the persistently low levels of mTOR, which were sufficient to maintain low cell proliferation or impact autophagy [41].

Focal adhesion kinase (FAK) also makes an important contribution to growth factor receptor and integrin signaling [47]. Studies focusing on esophagus squamous carcinoma linked pFAK to high mTOR S6K1 expression and consequently to the mTOR pathway. Investigations on FAK autophosphorylation in epithelial OC showed that while elevated levels of FAK were associated with distant and lymph-node metastasis, high levels of FAK were concurrently linked to improved OS [48], thus presenting an intriguing entity in gynecological cancer. Investigating ovarian clear cell carcinoma, Sato and colleagues found that phosphorylation of FAK was increased in spheroids, but not in adherent cells. This, in turn, provides a rationale for considering FAK inhibition as a supplement to current inhibition routes [49].

GDF-15 has already been associated with oncogenesis and worse outcomes in breast cancer and brain tumor models. Due to the unknown mechanism of action in tumor progression, subsequent investigations were also performed in ovarian cancer. It was shown that overexpression of GDF-15 contributed to the upregulation of MMP2, MMP9 and VEGF. This effect, however, could be inhibited by the use of inhibitors p38, MEK, and PI3K [44]. 

In the light of the impact of growth factors on endometrial cells, Subramaniam and colleagues [50] investigated cancer-associated fibroblasts (CAFs) in the vicinity of the EC cells. They reported that, compared to normal fibroblasts, CAFs secreted higher levels of macrophage chemoattractant protein (MCP)-1, interleukin (IL)-6, IL-8, RANTES, and vascular endothelial growth factor (VEGF). This suggests that fibroblasts can have a pro-tumorigenic effect on the progression of endometrial cancer, and PI3K/AKT and MAPK/ERK signaling may be critical regarding their maintenance [50]. Cell line studies have shown that inhibition of fibroblast growth factor receptor 2 (FGFR2), together with inhibition of mTOR, leads to a synergistic positive effect in EC.

The FGFR2 mutations are present in approximately 10% of EC and are mutually exclusive with KRAS mutations. FGFR2 is highly associated with PTEN loss, and a majority of tumors with FGFR2 mutations also carry PTEN mutations [51]. 

### 3.6. Genomic Aberrations Interacting with mTOR Signaling Pathway Activation 

A recent analysis of genomic data on endometrial cancer has identified the IGF-1/mTOR pathway in genome-wide association study (GWAS) and exome-Seq data as a relevant pathway, following the p53 pathway, which is the most relevant one [52]. The downstream targets of the IGF1 signaling, i.e., EIF2S1 and EIF2B5, have been associated with EC. In addition, according to findings based on the Human cancer genome atlas (HCGA), single nucleotide polymorphisms (SNPs) connected to PIK3CA, PIK3R1, and IGF1R are commonly found in EC [52]. In order to understand functional SNPs and target genes, Painter and colleagues carried out in silico fine mapping of patients with EC. They found that SNP rs2494737, a member of the PI3K/AKT/mTOR pathway located within AKT1, is activated in EC. The SNPs, however, also influence the silencer activity and affect YY1 siRNA, a positive regulator of AKT1. The expression of this SNP is therefore also related to the risk of EC development [53]. In a Taiwanese population, 50% of endometrial cancers displayed mutations in the PTEN gene. A smaller number of cases, however, showed aberrations in PIK3R1, AKT2, FOXO1, which contributed to the activation of the IL-7 signaling pathway [54].

Genetic analysis carried out in serous high-grade ovarian cancer shows that PI3KCA mutations are present in 18% of the cases and PTEN mutations in 7% of the cases. This is also true for mutations (PIK3CA) in endometrioid and mucinous carcinomas (20% of cases had mutations) as well as in clear cell tumors (46% of the case series had carcinomas). Activation of the AKT2 kinase was also observed in 40% of mostly high grade serous ovarian tumors. A simultaneous activation of AKT and mTOR occurred in 87% of ovarian tumors [55]. 

PI3K is at the modification level of the mTOR initiation cascade. Of importance are changes in Gab2 expression, which impact the EMT through the PI3K-Zeb1 pathway in OC. This mechanism is activated by the inhibition of E-cadherin expression [56]. Duckworth et al. then investigated the role of Gab2 expression in chemokine expression. They found that overexpression of Gab2 upregulated chemokine ligand 1 (CXCL1), CXCL2, and CXCL8. Interestingly, however, only pharmacological inhibition of IKKbeta, a target in NF kappa-B signaling, leads to the suppression of Gab2-induced chemokine expression. Other targets, among them PI3K, did not lead to the suppression of chemokine activity, thus showing that co-regulating the nuclear factor kappa B (NF-KB) and the mTOR pathways could be beneficial in OC [57]. Wang and colleagues [56] then further investigated the role of Zeb1 in the transcription process in ovarian cancer. Through the expression of the Gab2 protein, they found that if mutant Gab2 variants were present, the activation of PI3K and Shp2-ERK pathways was defective. Gab2 presence enhanced the expression of Zeb1, a factor involved in the epithelial-to-mesenchymal transition, cell migration and invasion, thus worsening the prognosis of the malignancy.

### 3.7. Epithelial-Mesenchymal Transition and mTOR Signaling 

Understanding the induction of EMT in gynecological cancers is important in terms of understanding metastasis mechanisms. Regardless of our knowledge that EMT changes lead to cell migration and invasion ability, the mechanism how mTOR interacts with EMT is unclear. A proposed trigger of EMT transformation is the Transforming Growth Factor beta (TGF-b). TGF-b activates AKT through PI3K and subsequently activates the mTORC1 cascade and its downstream targets of p70S6 and eukaryotic initiation factors. Studying the role of TGF-b, Chen and colleagues found that inhibiting the mTOR signaling leads to downregulation of pyruvate kinase M2 (PKM2), which is needed to induce EMT in cervical cancer cells [58]. EMT was studied in histologically high-grade OC. An analysis of cell lines and Western Blot revealed that the expression of PI3K, pAKT, and p-mTOR was decreased after γ-Glutamyl cyclotransferase (GGCT) knockdown. This resulted in decreased proliferation, clone formation, and migration, which led some authors to conclude that GGCT could be used as a biomarker or potential therapeutic target [59]. Forkhead Box P3 (Foxp3) was also examined for its function in carcinogenesis. The upregulation of Foxp3 showed a decrease of Ki-67 and cyclin-dependent kinases (CDKs), as well as downregulation of matrix metalloproteinase-2 (MMP-2) and urokinase-type plasminogen activator (uPA). In malignancies, MMPs and uPA are involved in the development of metastasis. Their role is defined by the inhibition of cell migration and invasion, and they inhibited mTOR signaling and NF-kB signaling [60]. These molecules can be involved as downstream actors (Figure 2) in connection with the enhanced Snail expression, which is associated with YB-1 mRNA expression [19]. 

### 3.8. RNA-Based Alterations Interacting with mTOR 

RNA-based alterations can be evaluated through long non-coding RNA (lncRNA) and microRNA (miRNA) changes. miRNA consists of short RNAs of 18–22 nucleotides with non-coding characteristics and regulates the expression of a variety of genes. miRNA regulates the gene expression by binding to the 3′-untranslated regions (3′-UTRs) of messenger RNA (mRNA) [61,62]. It is assumed that more than half of the mRNAs are regulated by miRNA, which was found to enhance degradation or inhibit post-transcriptional translation of mRNA. As approximately half of the miRNA genes are located in cancer-associated regions of the genome or at sites vulnerable to cancer-based aberrations, miRNAs are suggested to play an important role in carcinogenesis [61]. Investigating cervical cancer transfected with HPV 16, Nair and colleagues described a specific miRNA profile correlating with HPV16-transfected cervical cancer (downregulation of miR-1184, miR-377-3p, miR-136-5p, miR-218-5p, miR-4687-3p, miR-497-5p, and miR-5572 and upregulation of miR-21-5p, miR-429, miR-135b-5p, and miR-363-3p [63]). Analysis of the cancer genome atlas (TCGA) miRNA data further on identified a signature of miRNA for cervical cancer. Their risk scoring showed that an interplay of miR-3154 and miR-7-3 was correlated with shortened OS, and the miR-600 was significantly associated with improved OS [64]. Table 2 depicts the miRNAs identified for different gynecological cancer subtypes. In gynecological cancers, only a few miRNAs overlap due to the regulation of the mTOR pathway. These consist of the miR99 family (consisting of miR-99a and miR-100), which is present in all gynecological malignancies, and the miR-199 family, detected in endometrial and ovarian carcinoma. According to Torres et al., the three mTOR signature of miR-99a, miR-100 and miR199b is an miRNA signature that needs to be down-regulated in order to enable mTOR kinase upregulation [65]. 

Discoveries made over the last few years have identified long non-coding RNA (lncRNA), an important stakeholder in carcinogenesis signaling. Previously, lncRNAs were considered a transcriptional noise. This class of RNA, containing more than 200 nucleotides, is involved in the processes of translation, RNAsplicing and gene regulation [79,80]. In the understanding of pathological processes, lncRNA was given the role of a co-player influencing proliferation, apoptosis, cell cycle inference, migration, invasion, metastasis, and drug resistance [79]. A few lncRNAs have been identified in gynecological cancers. A systematic analysis of the literature showed that among currently identified lncRNAs, there are two overlapping lncRNAs in gynecological malignancies. These are MEG3 (maternally expressed gene 3) [81,82,83] and DLEU1 (deleted in lymphocytic leukemia 1) [84,85,86], which have been identified in all three gynecological cancers. MEG3 is a maternally imprinted gene functioning as a tumor suppressor. It is often found in normal tissue and is expressed only in some cancer tissue types. In EC, it was demonstrated that lncRNA MEG3 can be directly combined with PI3K, thus impacting tumor growth. Compared to normal tissue, endometrial and ovarian carcinomas showed high expression of lncRNA DLEU1 [84,86]. This overexpression has been reported to increase cell viability, proliferation, migration, and invasion. Subsequent studies have shown that changes in the expression of AKT1, p70S6K, rpS6, GSK3beta, signal transducer and activator of transcription 3 (STAT3), and B-cell lymphoma-extra large (BCl-xl) were correlated with changes of lncRNA, DLEU1, and mTOR in endometrial carcinoma [84]. A significantly low expression of lncRNA Cancer Susceptibility 2 (CASC2) was identified in ovarian carcinoma. The initiation factor eIF4A3 acted as a CASC2-binding protein [87].

### 3.9. Hypoxic Modulation of Carcinogenesis via mTOR Signaling 

Rapidly expanding tumors outgrow their vascular supply and as such become hypoxic. Hypoxic conditions enable the enhancement of tumor invasiveness. The process is mostly regulated by the expression of hypoxia inducible factors (HIFs) [88]. Investigating OC, Gomez-Roman et al. have found that HIFs, in order to become invasive, need to be induced by Rab25, a small GTPase. Rab25 functions as a downstream target of p70S6K. The protein p70S6K was overexpressed in cells with levels of Rab25, which were overexpressed as well. This indicates that the activation of the mTOR signaling, specifically the downstream targets of p70S6K, is needed to induce HIF [88]. Research on CC showed that the induction of HIF-1alpha is controlled by Pak4, another p21-activated kinase of the serine/threonine kinase family affecting the Rho-related GTPases, such as Rac and Cdc42, in hypoxic conditions. In vitro experiments showed that the knockdown of Rac4 attenuated the expression of HIF-1alpha. The process of Pak4 control was modulated by AKT and the downstream modulation of the translation inihibitor 4E-BP1 [89]. The regulation of HIF, however, is not yet fully understood, and is most likely dependent not only on the regulation through GTPases. Investigation into the protein kinase C (PKC) family showed that, in cancer cells, PKC accumulation is dependent on HIF-1a. Tests conducted in three different cell lines (cervical and prostate cancer as well as fibrosarcoma) led to the hypothesis that tumor-caused hypoxia most likely induces PKC isoform-dependent HIF-1a accumulations. Cell line studies have shown that the HIF1alpha activation was initiated by the AKT-mTOR and NF-kB cascade [90,91].

Hypoxia has been interconnected with autophagy [71]. Autophagy, as a cytoprotective process, represents an important step in carcinogenesis as it facilitates cell survival and accompanies a multistep process to degrade cell components. Dysregulation of this process contributes to tumor progression [13]. Autophagy, which is lower in cancerous than in normal cells, has been observed in malignant cells and cells with malignant potential [72]. An evaluation of the levels of miR155 in cervical and nasopharyngeal cancer shows that if members of the mTOR pathway (RHEB, RICTOR and RPS6KB2) are activated, the levels of miR155 are lowered and autophagy is decreased. This is most likely a consequence of miR-155 impacting PDK1, which is an upstream kinase of AKT that can activate AKT. An attenuation of the pathway leads to an increase in autophagy in the in vitro models. This had an impact on cell proliferation and G1/S cell cycle progression [71]. Furthermore, due to the impact of PTEN, miR-205 induced autophagy was attenuated. [67]. Processes interconnecting authophagy dysregulation linked to the mTOR pathway have been observed on different levels. In OC, the expression of DIRAS 1 and 2, GTP-ases, leads to the inhibition of S473 AKT1 and S448 mTOR phosphorylation. DIRAS 1 and 2 expression was downregulated and correlated with lower DFS and OS [92]. Further studies investigating ARHI (also known as DIRAS3) in OC found that it induces autophagy by blocking mTOR and PI3K signaling. Further, 60% of OC samples in that study showed downregulated expression of DIRAS3. However, this study also showed that ARHI overexpression leads to autophagic cell death, but enabled dormant carcinoma cell survival [93].

## 4. Conclusions

YB-1 interacts with mTOR mostly through the nuclear YB-1. The data demonstarte that dual inhibition of mTOR and YB-1 shows promising decreases in carcinogenic activity. The downstream effects in gynecological cancer (Figure 2) show an important interconnection of mTOR with YB-1 activity. However, in gynecological cancers, there have only been a few identified targets of YB-1 action until now.

Our imperfect understanding of the interplay of YB-1 and mTOR is even more pronounced in endometrial cancer. While we have observed that YB-1 influences VEGF, our observation of the communication of cancer-associated fibroblasts (CAFs) with the overexpression of interleukin (IL-6), Il-8, RANTES, and VEGF needs to be further evaluated in light of data showing the involvement of YB-1. The role of growth factors, especially VEGF, with the mTOR pathway through CAF stimulation has yet to be studied in ovarian and cervical cancers. Furthermore, we point towards evidence of the presence of an AKT/mTORC2-mediated process in the hypoxic environment. Especially as YB-1 is implicated to act upon AKT expression, the process and interconnection with mTORC2 must be further investigated. All gynecological cancers have been reported to show an overexpression of HIF in a hypoxic environment, but data suggest that HIF enables specific isoforms of the PKC family to accumulate. As the cascade continues, this leads to an mTORC2-mediated effect currently reported only in cervical cancer [94,95]. Therefore, a further inquiry to be opened by this review is the role played by hypoxia downstream in the mTORC2 cascade. The question arises whether mTORC2 signaling plays a role in all gynecological cancer signaling procedures. Interestingly, research elucidated that the mTOR pathway probably results in alterations due to YB-1 activity and is most likely not impacted by eukaryotic initiation translation factors.

## Figures and Tables

**Table 1:** Ongoing clinical trials * involving mTOR in gynecological cancer.

Target	Therapeutic Agent	Carcinoma	Trial Drug Design	Reg. Nr.
mTORC1	everolimus	EC	Everolimus (RAD001) and Letrozole or Hormonal Therapy	*NCT02228681*
		Atypical hyperplasia or FIGO stage IA EC	Levonorgestrel-Releasing Intrauterine System with or without Everolimus	NCT02397083
		OC, EC, CC	Patients with alterations in PIK3CA, PIK3R1, AKT1, AKT2, mTOR, RICTOR, RAPTOR genes, or with TSC1, TSC2 or PTEN loss for maintenance therapy	NCT02029001
	everolimus temsirolimus	Advanced OC	Vemurafenib in Combination with Everolimus or Temsirolimus	*NCT01596140*
	sirolimus	Stage II-IV OC	The effects of TRICOM vaccine with sirolimus	*NCT01536054*
mTORC1/2	Vistusertib (AZD2014)	HR positive EC	AZD2014 and anastrozole vs. anastrozole alone	*NCT02730923*
	Sapanisertib MLN0128	EC and other solid tumors	Single experimental arm: bevacizumab and MLN0128	NCT02142803
	Sapanisertib MLN0128	EC, OC	Patients with mTOR mutation receive sapanisertib as a single experimental arm	NCT02465060
AKT inhibitors	miransertib ARQ 092	OC, CC, EC	ARQ 092 + paclitaxel or ARQ 092 + anastrozole	NCT02476955
PI3K inhibition	Copanlisib Hydrochloride	CC, OC, EC	Patients with a PI3K or a PTEN mutation receive copanlisib	NCT02465060
	GSK2636771	CC, OC, EC	Patients with a PTEN mutation/expression/loss	NCT02465060
Dual PI3K/mTOR inhibition	Gedatolisib PF-05212384	EC and other solid tumors	Single experimental arm: palbociclib and gedatolisib	NCT03065062
mTORC1/2 vs. AKT inhibition	vistusertib AZD2014 vs. capivasertib AZD5363	Recurrent EC or OC	(olaparib, vistusertib) vs. (olaparib, capivasertib)	*NCT02208375*
Dual Akt/ERK inhibition	ONC201	Recurrent or metastatic EC	Single experimental arm: ONC201 treatment	NCT03099499

* accurate data as of 3rd January 2020. Abbreviations: EC: endometrial cancer, OC: ovarian cancer, CC: cervical cancer, HR: hormone receptor; mTORC1: mammalian target of rapamycin complex 1, AKT: protein kinase B, PI3K: Phosphoinositide 3-kinase, mTOR: mammalian target of rapamycin.

**Table 2:** Overview of miRNA molecules identified interacting with gynecological cancers through the PI3K/Akt/mTOR pathway.

Type of Investigated Cancer	miRNA	Component	Impact of Aberration on Signaling
Endometrial cancer	miR-101 [66]	mTORC1/mTORC2	Downregulation leads to mTOR upregulation
	miR-205 [67]	PTEN expression regulation	Low levels of miR-205 lead to reduced pmTOR and pAKT expression
	miR99 family [65,68]	PI3K through direct target IGF/IGFR	Low levels of expression correlated with better tumor differentiation
	miR-199a-3p [69]	mTORC1/mTORC2	Upregulation inhibits tumor cell proliferation through negative regulation of mTOR expression
	miR-199b [65]	mTOR kinase	High expression in better differentiated EC
	miR-152 [70]	Impacts RICTOR and the mTORC2-AKT cascade	Downregulation enables CpG island hypermethylation
Cervical cancer	miR-155 family [71,72]	3′UTR of PDK1	Enables PDK1 the activation of the mTOR pathway when under expression
	miR-99 family [73]	PI3K through the direct target IGF/IGFR	Upregulation directly negatively regulates mTOR expression
	miR-634 [74]	Direct binding to 3ÚTR of mTOR	Upregulation represses mTOR expression
	miR-338 [13]	No direct information—downregulation of PI3K and AKT, upregulation of pmTOR and p70S6	Downregulation inhibits autophagy by targeting ATF2
Ovarian cancer	miR-206 [75]	c-Met	Downregulation impacts estrogen receptor as a direct target, enables growth and invasion in EOC
	miR-130a [76]	TSC1	Downregulation enables enhanced mTOR activity
	miR-199a [77]	mTOR	Upregulation blocks mTOR expression
	miR-100 [78]	FRAP1/mTOR	Down regulation leads to enhanced mTOR pathway activity
	MiR-206 [75]	c-Met	Direct activation of downstream AKT/mTOR signaling pathway

mTORC1- mammalian target of rapamycin complex 1; PTEN - Phosphatase and tensin homolog; PI3K- Phosphoinositide 3-kinases; IGF/IGFR: Insulin growth factor/receptor, RICTOR: rapamycin-insensitive companion of mammalian target of rapamycin, UTR: untranslated region, PDK1: Pyruvate Dehydrogenase Kinase 1, TSC1: Tuberous sclerosis protein, FRAP1/mTOR: FK506-binding protein 12-rapamycin-associated protein 1/ mammalian target of rapamycin.
